# Multiple Developmental Defects in *sox11a* Mutant Zebrafish with Features of Coffin-Siris Syndrome

**DOI:** 10.7150/ijbs.47510

**Published:** 2020-10-03

**Authors:** Shaoting Jia, Xingxing Wu, Yunya Wu, Xuefan Cui, Binbin Tao, Zuoyan Zhu, Wei Hu

**Affiliations:** 1 State Key Laboratory of Freshwater Ecology and Biotechnology, Institute of Hydrobiology, Chinese Academy of Sciences, Wuhan 430072, China.; 2 Qingdao National Laboratory for Marine Science and Technology, Qingdao 266237, China.; 3 University of Chinese Academy of Sciences, Beijing 100049, China.

**Keywords:** *sox11*, zebrafish, Coffin-Siris, CRISPR/Cas9

## Abstract

A previous study suggested that human Coffin-Siris syndrome is related to the mutation of *SOX11*. Since the homozygous *SOX11* mutant mice died soon after birth, no suitable model was available for the study of the pathogenic mechanism of Coffin-Siris syndrome. To solve this problem, we generated two viable homozygous zebrafish mutants, *sox11a*^*m/m*^ and *sox11b^m/m^*. We found that the *sox11a^m/m^* mutant possessed Coffin-Siris syndrome features. The *sox11a^m/m^* mutants exhibited growth deficiency from 3.3 hpf embryos to adulthood. Furthermore, the *sox11a^m/m^* mutant also displayed microcephaly, narrow pupillary distance, achondroplasia, and bone deformity in adults. Growth deficiency could be rescued by the injection of *sox11a* mRNA at the one-cell stage. In addition, the expression levels of genes related to cartilage and bone were downregulated in the *sox11a^m/m^* mutant, indicating that *sox11a* mainly affected the growth and development of zebrafish by regulating the expression of genes related to skeletal development. Our results indicate that *sox11a^m/m^* mutant zebrafish offered a potential model system to help with the search for pathogenic mechanisms of human Coffin-Siris syndrome.

## Introduction

There are seven members in the sox family, namely soxA, soxB1, soxB2, soxC, soxD, soxE, soxF, and soxG 1. The functional domain of the SOX protein is an HMG domain consisting of three α helices that can bind to the ATTGTT sequence or other related sequences 2,3. Therefore, all sox family members are transcription factors. *sox11* comes from the soxC sub-family, which also includes *sox4* and *sox12* 4. Studies have illustrated that *sox11* is involved in multiple biological processes, including neurogenesis, organogenesis, craniofacial, and skeletal development 5,6.

Coffin-Siris syndrome is a disease that has affected humans for a long time, and most of the patients show retardation of motor and language expression abilities and bone development, besides small body size and low intelligence level 7-9. Clinical data has showed that the disease is related to *SOX11* mutation. Hempel et al. reported that the cause of Coffin-Siris syndrome in 10 patients was associated with *SOX11* deletion or mutation 7. However, the specific pathogenic mechanism of Coffin-Siris syndrome is not clear. The function of *Sox11* is relevant to tissue remodeling, and *Sox11* mutant mice have been shown to develop symptoms of congenital cyanosis, congenital heart disease, cranial hypoplasia, and non-cranial dysplasia, besides embryo death immediately after birth 10. However, the difficulty of obtaining viable homozygote mutants hinders the study of the function and underlying mechanisms of *Sox11* in mammals.

Zebrafish are widely used as model organisms in vertebrate development research and disease model. There are two* sox11* genes in zebrafish including *sox11a* and *sox11b*. However, the role and potential mechanisms of *sox11a/b* in zebrafish remain largely unknown, and there is no *sox11a/b* mutation model in zebrafish. Therefore, our study aimed to further elucidate the mechanism of Coffin-Siris syndrome diseases and establish appropriate disease models by revealing and comparing *sox11a/b* functions in zebrafish development. For this, we obtained viable *sox11a^m/m^* and *sox11b^m/m^* mutants for the first time using the CRISPR/Cas9 system. We found that *sox11a^m/m^* mutant zebrafish exhibited reduced size and abnormal cartilages and bones, with smaller heads and lower pupillary distances compared to non-mutant zebrafish. However, there were no obvious differences between the *sox11b^m/m^* mutants and WT individuals. Therefore, only *sox11a^m/m^* mutant zebrafish can be used to explore the pathogenic mechanism of Coffin-Siris syndrome.

## Materials and Methods

### Zebrafish maintenance

In this study, AB strain zebrafish were used and maintained according to the zebrafish book 11, raised in recirculation systems at 28 °C and under a 14 h: 10 h light: dark cycle. All zebrafish experiments were performed according to the Guiding Principles for the Care and Use of Laboratory Animals approved by the Institute of Hydrobiology, Chinese Academy of Sciences.

### Generation of *sox11a* and *sox11b* mutant zebrafish lines and double mutant lines

Disruptions of *sox11a* and *sox11b* in zebrafish were accomplished via CRISPR/Cas9 technology. Zebrafish *sox11a* and *sox11b* sgRNA were designed using tools provided on a website (http://www.crisprscan.org/?page=sequence), and the target sites were GGTCGCTTTATGTGTC NGG and GGGTCGTTTGATGTGGC NGG, respectively. The primers were designed according to the pT7gRNA and target sites. pT7gRNA was used as a template to synthesize the gRNA *in vitro* transcription template using PCR. The forward and reverse primers of the two target sites were shown in Figure S1. The transcription kit (Invitrogen, USA) was used for *in vitro* gRNA transcript and the gRNA was purified using the gRNA purification kit (QIAGEN, Germany). Cas9 mRNA was obtained using an *in vitro* transcription kit (Invitrogen, USA) with the linearizing Cas9 plasmid as template and purified by ammonium acetate precipitation. The gRNA and Cas9 mRNA were diluted to 50 ng/µL and 500 ng/µL, respectively, and then mixed according to a 1:1 ratio with a small amount of phenol red as an indicator. The prepared sample was injected into the animal pole of the embryos at the one-cell stage and then cultured in 0.3 × Danieau' s buffer for P0 generations. Then, P0 generation adult fish were crossed with WT to produce the F1 generation. The screened heterozygote F1 generation was self-crossed to produce the F2 generation, from which the homozygote mutation could be identified. The homozygote F2 generation mutation was then self-crossed to produce the F3 generation, which was used in the following experiments.

The *sox11a^m/+^sox11b^m/+^* double heterozygous genotype mutants were produced by crossing the *sox11a^m/m^*mutants with the *sox11b^m/m^* mutants. We expected to obtain the *sox11a^m/m^sox11b^m/m^* double mutants by genotyping offspring which was produced by *sox11a^m/+^sox11b^m/+^* double heterozygous self-crossing. In theory, the following genotype mutants could be produced as shown in Figure S7. However, the number of the *sox11a^m/m^sox11b^m/m^* double mutants was zero after genotyping 46 offspring. Then we picked up three groups including *sox11a^m/m^sox11b^m/+^*self-crossing, *sox11a^m/+^sox11b^m/m^*self-crossing and crossing the *sox11a^m/+^sox11b^m/m^*mutant with the *sox11a^m/+^sox11b^m/+^* mutant to produce the double mutants.

### Real-time quantitative PCR (qPCR)

Total RNA from zebrafish tissues and embryos was extracted with TRIzol (Invitrogen, USA) according to the manufacturer's instructions. The obtained RNA was analyzed by agarose gel electrophoresis, and its concentration was measured using a spectrophotometer (Thermo, USA). Then, 1 µg of RNA was removed for DNA removal, and a kit (Toyobo, Japan) was used for reverse transcription. cDNA was obtained using the ReverTra Ace reverse transcriptase kit (Toyobo, Japan) and random primers. The fluorescence quantitative PCR assay was performed using the SYBR green mix (Toyobo, Japan) according to the manufacturer's instructions: 2×SYBR Green real-time master mix, 10 µL; cDNA, 2 µL; F, 0.4 µL; R, 0.4 µL; H_2_O, 7.2 µL. The PCR amplification conditions were: 95 °C for 3 min; 45 cycles × (95 °C for 15 s; 55 °C for 20 s; 72 °C for 30 s); 65 °C for 0.06 s; 95 ℃, 0.5 s. The primers were listed in Figure S1.

### Assessment of growth parameters

The body length of the zebrafish was measured based on photographs using the ImageJ software. Body weight was determined using an electronic analytical balance (Shimadzu, Japan) after the individual's body was dried using filter paper.

### Histological analysis

We observed adult zebrafish muscle development and embryo brain growth using paraffin sections. The samples were fixed in Bouin's fixative overnight. After dehydration, penetration, and wax immersion, the samples were embedded in paraffin wax. The samples were then sectioned into 6-μm slices and stained using the hematoxylin and eosin method. The images were taken using a microscope (OLYMPUS, Japan).

### Immunofluorescence staining

Whole mount muscles of zebrafish larvae were analyzed by immunofluorescent staining. For this, the samples were immersed in 4% PFA overnight at 4 °C. The fixed samples were washed with PBS every 5 min three times and permeabilized for 30 min in 0.1% Triton PBS. The samples were then stained with phalloidin (1:500) overnight at 4 °C. The nuclei were stained with DAPI (1:1000). After washing three times with PBS, the samples were sealed with mounting medium (Vectorlabs, USA) and the images were taken using a confocal microscope (Leica, Germany).

### Alcian blue staining

To observe the cartilage development of *sox11a^m/m^* mutant zebrafish, the samples were stained with Alcian blue. The 5-day post fertilization (dpf) zebrafish larvae were fixed in 4% PFA overnight at 4 °C. The samples were then transferred into 70% methanol. The samples were stained in an Alcian blue buffer (35 mL 100% MeOH, 4 mg MgCl_2_, 10 mg Alcian blue, 50 mL final volume) for 4 h after incubating the samples in 50% ethanol for 10 min on a decolorizing rocker. The samples were washed in PBST every 5 min four times before rinsing in rinsing buffer (30% H_2_O_2_ 5 mL, 1% KOH 25 mL, and 20 mL ddH_2_O, 50 mL final volume). Then, the fish body was digested with 1 mg/mL trypsin (100 mg trypsin, 100 mL 60% saturated sodium borate) near transparent after washing the samples in PBST every 5 min four times. Images were taken after washing the samples in PBST every 5 min twice.

### Micro-CT

For this procedure, a *sox11a^m/m^* mutant and a WT zebrafish of the same age were used. The body surfaces were dried using filter paper after anesthesia with MS222. Then, the bodies were wrapped with the bubble film matching the instrument and placed on the appropriate sample table before the scanning was carried out. When the scanning was completed, image reconstruction and processing was performed.

### Whole-mount *in situ* hybridization

Digoxigenin-labeled probes against *sox11a*, *sox11b*,* and1*, *crtap*, *sec23a* and* col10a1a* were generated by PCR using the primer pairs shown in Figure S1 with an added T7 polymerase binding site and synthesized using a T7 *in vitro* transcription kit (Invitrogen, USA). Whole-mount *in situ* hybridization was performed according to the method of Thisse et al. 12, and the images were taken with a Nikon digital camera.

### Rescue experiment

The *sox11a* and *gfp* cDNA sequences were cloned into pcsII vectors. The plasmids were linearized using NotI (NEB, USA). Then, the linearized vector was transcribed using the Sp6 mMessage mMachine kit (Invitrogen, USA) and purified with the RNA purification kit (Sigma, USA) according to the manufacturer' s instructions. The primers are listed in Figure S1. The samples were injected into the embryos at the one-cell stage at a concentration of 150 ng/µL.

## Results

### Conservation and expression patterns of *sox11a* and *sox11b* in zebrafish

Two *sox11* genes, *sox11a* and *sox11b,* occur in zebrafish. The amino acid sequence homology analysis results are shown in Figure S2. The BLASTP results showed that the similarity between zebrafish *sox11a* and its homologous gene in medaka, mouse, rat, and human was 74.25%, 58.44%, 58.29%, and 58.5%, respectively. The similarity between zebrafish *sox11b* and its homologous gene in medaka, mouse, rat, and human were 67.46%, 58.6%, 58.6%, and 81.43%, respectively. This indicates that both genes are evolutionarily conserved.

**Figure 1:**
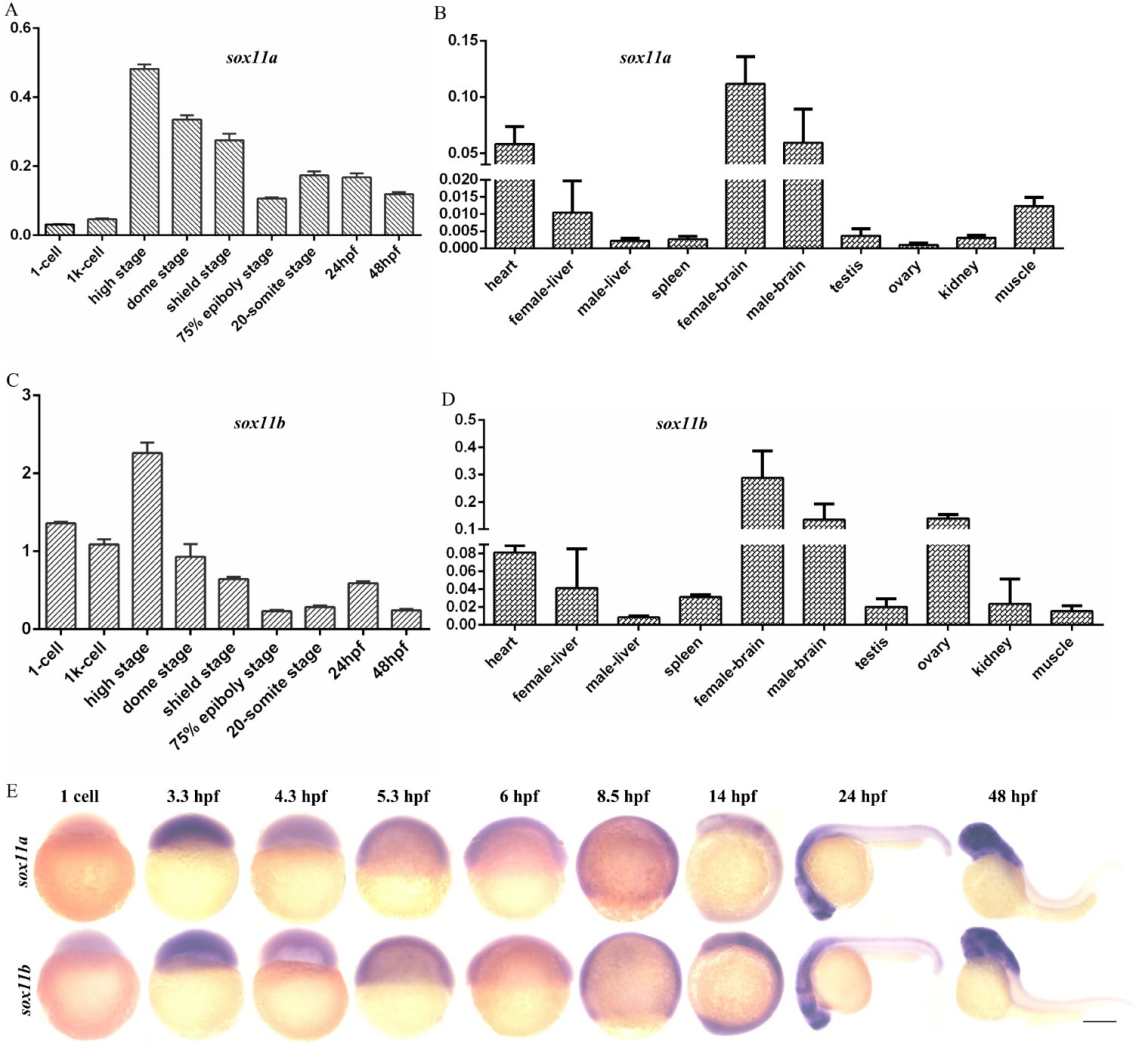
The expression patterns of *sox11a* and *sox11b* in zebrafish. **A:** qPCR analysis of zebrafish *sox11a* expression during early embryonic development. **B:** qPCR analysis of zebrafish *sox11a* expression in different tissues. **C:** qPCR analysis of zebrafish *sox11b* expression during early embryonic development. **D:** qPCR analysis of zebrafish *sox11b* expression in different tissues. The qPCR data were analyzed by 2^-∆CT^ method and *ꞵ-actin* was chosen as reference gene. **E:** The expression levels of *sox11a* and *sox11b* during early embryonic development by whole-mount *in situ* hybridization.

The expression levels of *sox11a* and *sox11b* in embryos of different stages and in different tissues of WT zebrafish were analyzed. The results indicated that *sox11a* and *sox11b* were expressed during various periods of embryonic development, with the highest expression in the high stage and the expression level gradually decreasing as the embryo developed (Fig. 1A, C). The mRNA expression pattern in adult tissues was that *sox11a* and *sox11b* mRNAs were mainly detected in the heart, brain, liver, muscle, and other tissues, with the highest levels in the brain (Fig. 1B, D). The expression of *sox11a* and *sox11b* was analyzed by whole-mount *in situ* hybridization (Fig. 1E).

### Generation of *sox11a* and *sox11b* mutants by CRISPR/Cas9

**Figure 2:**
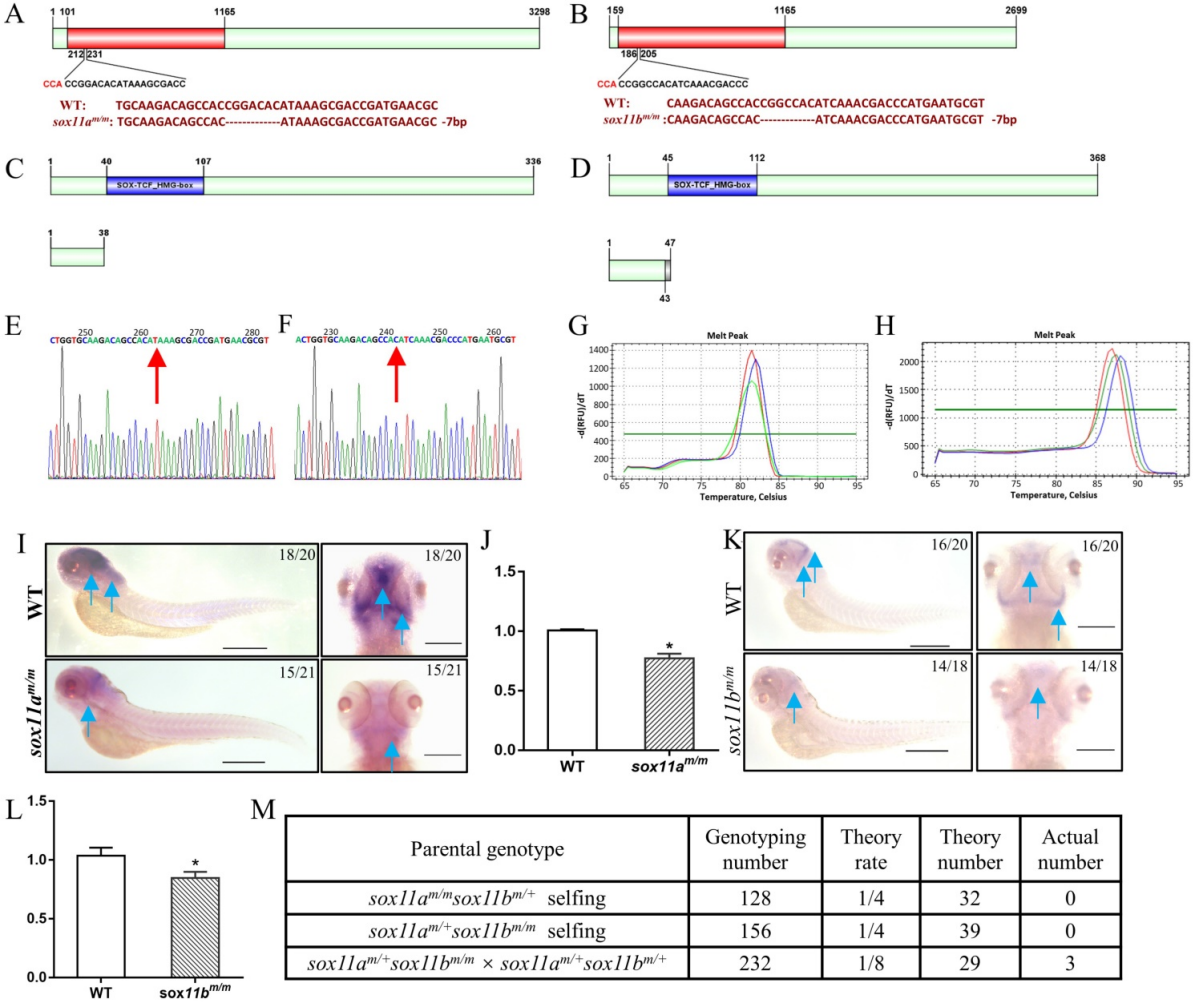
Establishment of *sox11a^m/m^* and* sox11b^m/m^*mutant line via CRISPR/Cas9 technology in zebrafish. **A:** The location of the sgRNA targeting sites on zebrafish *sox11a*. **B:** The location of sgRNA targeting sites on zebrafish *sox11b*. **C:** Schematic representation of the putative peptides of WT and the mutated *sox11a* peptides from the targeted allele. **D:** Schematic representation of the putative peptides of WT and the mutated *sox11b* peptides from the targeted allele. **E:** Confirmation of *sox11a* homozygote mutant by sequencing. **F:** Confirmation of *sox11b* homozygote mutant by sequencing. **G:** Screen of homozygote mutant by high resolution melting curve method. (red line, homozygote; green line, heterozygote; blue line, WT.) **H:** Screen of homozygote mutant by high resolution melting curve method. red line, homozygote; green line, heterozygote; blue line, WT. **I:** Whole-mount *in situ* hybridization of WT and *sox11a^m/m^* mutant. Antisense probes against *sox11a* were visualized at 4 dpf. Scale bar, 200 µm. **J:** The expression level of *sox11a* in WT and *sox11a^m/m^* mutant by qPCR. **K:** Whole-mount *in situ* hybridization of WT and *sox11b^m/m^* mutant. Antisense probes against *sox11b^m/m^* were visualized at 24 hpf. Scale bar, 200 µm. **L:** The expression level of *sox11b* in WT and *sox11b^m/m^* mutant by qPCR. The qPCR data were analyzed by 2^-∆∆CT^ method and *ꞵ-actin* was chosen as reference gene (*P<0.05). **M:** The generation of *sox11a^m/m^sox11b^m/m^* double mutant.

To further elucidate the function of *sox11* in zebrafish *in vivo*, *sox11a* and *sox11b* mutants were generated via CRISPR/Cas9. Two *sox11a* mutant lines were obtained, including a 7-bp deletion and a 11-bp deletion, which had a consistent phenotype (Fig. 2A, S3A-C). The F3 generation 7-bp deletion homozygote mutant line was used for subsequent experiments marked as *sox11a^m/m^*. The *sox11b* mutant line included a 1- bp deletion and a 7-bp deletion in two lines, which also had a consistent phenotype (Fig. 2B, S3D-F). The F3 generation 7-bp deletion homozygote mutant line was used for subsequent experiments marked as *sox11b^m/m^*.

The *sox11a^m/m^* homozygote mutant was confirmed by sequencing and screened using the high-resolution melting curve method, and the results are listed in Figure 2E, G. The amino acid sequence analysis of the *sox11a^m/m^* mutant line showed that it would produce a frameshift mutation and the translated amino acid sequence would terminate in front of the SOX-TCF_HMG-box domain (Fig. 2C). The whole-mount *in situ* hybridization results showed an obvious decrease in *sox11a* expression in the *sox11a^m/m^* mutant (Fig. 2I). And the qPCR results also indicated the expression level of *sox11a* in *sox11a^m/m^* mutant was lower than WT (Fig. 2J).

The *sox11b^m/m^* homozygote mutant was also checked using the high-resolution melting curve method (Fig. 2F, H). The translated amino acid sequence of the *sox11b^m/m^* mutant would produce a frameshift mutation and terminate ahead of the SOX-TCF_HMG-box domain (Fig. 2D). An apparent lower *sox11b* level in the *sox11b^m/m^* mutant could be found by whole-mount *in situ* hybridization (Fig. 2K). And the qPCR data also indicated the expression level of *sox11b* in *sox11b^m/m^* mutant was lower than WT (Fig. 2L).

In addition, we have tried to generate *sox11a^m/m^sox11b^m/m^* double mutant by three groups including: *sox11a^m/m^sox11b^m/+^*self-crossing, *sox11a^m/+^sox11b^m/m^*self-crossing and crossing the *sox11a^m/+^sox11b^m/m^*mutant with the *sox11a^m/+^sox11b^m/+^* mutant. However, only three adult *sox11a^m/m^sox11b^m/m^* double mutants were confirmed after genotyping 516 offspring from the three groups which deviated from the law of independent assortment. The detailed information of this result could be found in Figure 2M. In conclusion we failed to generate a double mutant family since the three *sox11a^m/m^sox11b^m/m^* double mutants couldn't produce offspring.

### *sox11a^m/m^* mutant zebrafish displayed growth and developmental delay while *sox11b^m/m^* mutant development normally

**Figure 3:**
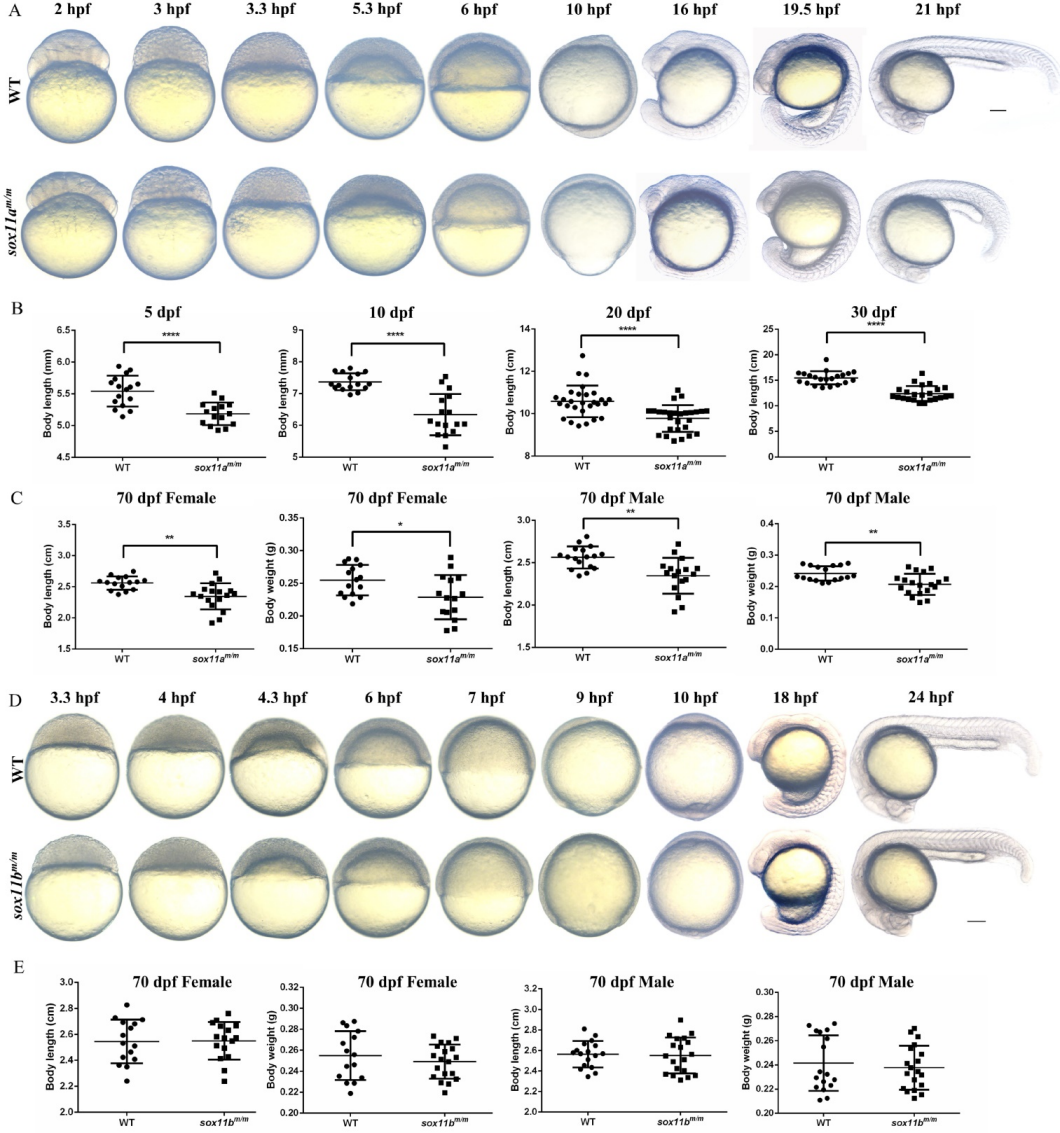
The *sox11a^m/m^* mutant exhibit growth retardation while the *sox11b^m/m^* mutant developed normally. **A:** The morphology of WT and *sox11a^m/m^* mutant larvae from 2 hpf to 21 hpf. Scale bar, 200 µm. **B:** The body length of *sox11a^m/m^*mutants was significantly shorter than that of WT from 5 dpf to 30 dpf (****P<0.0001). **C:** The 70 dpf female body length and body weight of *sox11a^m/m^* mutants were significantly smaller than that of WT. And the 70 dpf male body length and body weight of *sox11a^m/m^* mutants were also significantly smaller than that of WT (*P<0.05, **P<0.01). **D:** The morphology of WT and *sox11a^m/m^* mutant larvae from 3.3 hpf to 24 hpf. Scale bar, 200 µm. **E:** There were no significant difference between the 70 dpf female or male *sox11b^m/m^* mutants and their respective WT counterpart regarding body length and body weight.

First, the early embryo development process was observed by comparing *sox11a^m/m^* mutants with WT embryos. We found that *sox11a^m/m^* mutant embryos began to present some delay compared with WT embryos from 3.3 h post fertilization (hpf). This phenomenon was maintained throughout early embryo development. At 10 hpf, the WT embryos had developed to 100% epiboly stage, while the *sox11a^m/m^* mutant embryos fell behind. At 21 hpf, the *sox11a^m/m^* mutant embryo tail was clearly shorter than that of WT embryos (Fig. 3A).

Then, juvenile fish development was analyzed by measuring the body length of *sox11a^m/m^* mutants and WTs bred under the same conditions. Four points were chosen for statistical analysis including 5 dpf, 10 dpf, 20 dpf, and 30 dpf (Fig. 3B). The results indicated that the body length of *sox11a^m/m^* mutants was significantly smaller than that of WTs (****P<0.0001).

The 70 dpf adult zebrafish were separated into males and females before analysis. Then, the body length and body weight were measured and counted for each group separately (Fig. 3C). The results suggested that the body lengths of male and female *sox11a^m/m^* mutants were significantly lower than those of male and female WTs, respectively (**P<0.01). The body weight of female* sox11a^m/m^* mutants was lower than that of WT females (*P<0.05), as was that of male* sox11a^m/m^* mutants compared to WT males (**P<0.01).

In addition, the *sox11b^m/m^* mutant zebrafish was also observed and analyzed. The developmental process of the *sox11b^m/m^* mutant embryo was in accordance with that of WT during the early embryo development stage (Fig. 3D). Subsequently, we measured the body length and body weight of the adult zebrafish, including males and females (Fig. 3E). The results showed that there was no significant difference in body length between males and females. The body weight of the *sox11b^m/m^* mutant and WT showed no difference either (*P*>0.05).

### *sox11a^m/m^* mutant zebrafish showed microcephaly, normal muscle and curved spine

**Figure 4:**
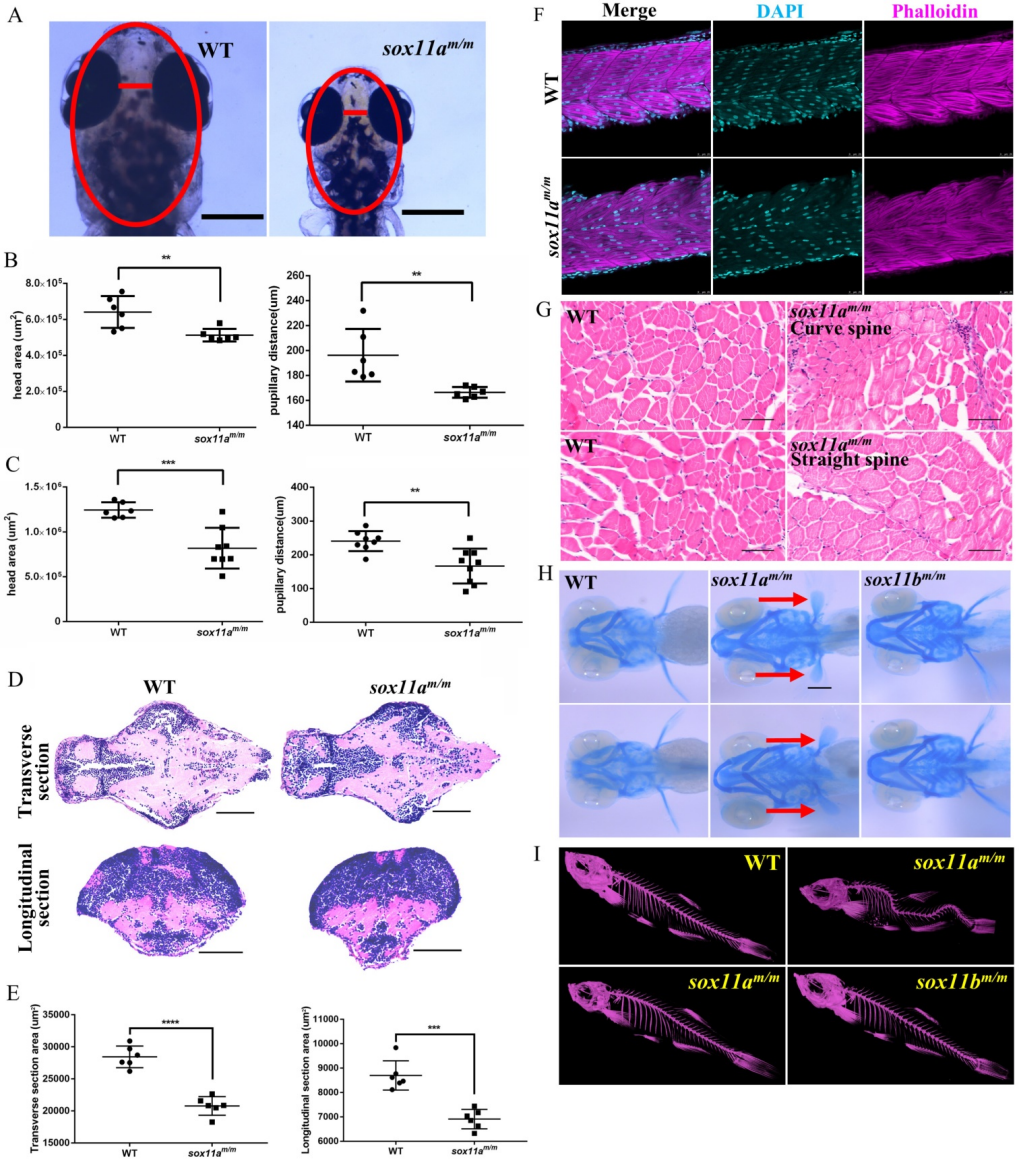
The *sox11a^m/m^* mutant zebrafish exhibit microcephaly, normal muscle and curved spine. **A:** The head area and pupillary distance of *sox11a^m/m^* mutant were significantly smaller than WT. Scale bar, 500 µm. **B:** The head area and pupillary distance of *sox11a^m/m^*mutants were significantly smaller than that of WT in 5 dpf (**P<0.01). **C:** The head area and pupillary distance of *sox11a^m/m^*mutants were significantly smaller than that of WT in 10 dpf (**P<0.01, ***P<0.001). **D:** The brain section of *sox11a^m/m^*mutant at 5 dpf was obviously smaller than WT including the transverse and longitudinal sections. Scale bar, 100 µm. **E:** Statics of the area of the brain section. **F:** The myoblast fusion in 48 hpf larvae. (Cyan: DAPI; Magenta: Phalloidin.) Scale bar, 25 µm. **G:** Arrangement of muscle fibers of muscles in adult zebrafish.* sox11a^m/m^*Curve spine: the mutant with curved spine; *sox11a^m/m^*Straight spine: the mutant without curved spine. Scare bar, 50 µm. **H:** The pectoral fin development of *sox11a^m/m^* mutant zebrafish obviously lagged behind that of WT and *sox11b^m/m^* mutant. **I:** Micro-CT of adult zebrafish. Some* sox11a^m/m^*mutant had curved spine compared with WT while all the *sox11b^m/m^*mutants were normal.

*sox11a* is essential not only for growth but also for head development. The head area and pupillary distance of the embryos were measured using Image J, based on the scale bars in the photographs (Fig. 4A). The results showed that both the head area and pupillary distance of the* sox11a^m/m^* mutants at 5 dpf were significantly smaller than those of WTs (Fig. 4B, **P<0.01). At 10 dpf, the head area of the* sox11a^m/m^* mutants was much smaller than that of the WTs (***P<0.001), as was the pupillary distance (Fig. 4C, **P<0.01). The paraffin section indicated the brain of *sox11a^m/m^* mutant was smaller than WT including the transverse and longitudinal sections (Fig. 4D, E ***P<0.001, ****P<0.0001). These data conformably indicated that the *sox11a^m/m^* mutants had microcephaly defects.

To investigate why *sox11a^m/m^* mutants display developmental delay, the myoblast fusion of the larvae was first examined. Larvae at 48 hpf were stained with phalloidin, and the results showed no significant difference between *sox11a^m/m^* mutants and WT larvae regarding the myoblast fusion (Fig. 4F). Furthermore, the muscles of adult zebrafish were studied using paraffin sections. The results showed that there was no obvious difference in muscle development among *sox11a^m/m^* mutant with curve body, *sox11a^m/m^* mutant with straight body and WTs (Fig. 4G).

To further determine whether the *sox11a^m/m^* mutant growth defect was caused by the skeleton, Alcian blue staining was conducted to observe cartilage development (Fig. 4H). We noted that the pectoral fin development of *sox11a^m/m^* mutant zebrafish was clearly slower than that of WTs and *sox11b^m/m^* mutants. Then, the bone development of adult zebrafish was checked using micro-CT. Figure 4I shows that some *sox11a^m/m^* mutants presented curved spines, whereas the bones of WTs and *sox11b^m/m^* mutants were normal. A total of 33% (±2%) of *sox11a^m/m^* mutants presented severely curved spines (Fig. S5, ****P<0.0001).

### The *sox11a^m/m^* development delay could be rescued by *sox11a* mRNA

**Figure 5:**
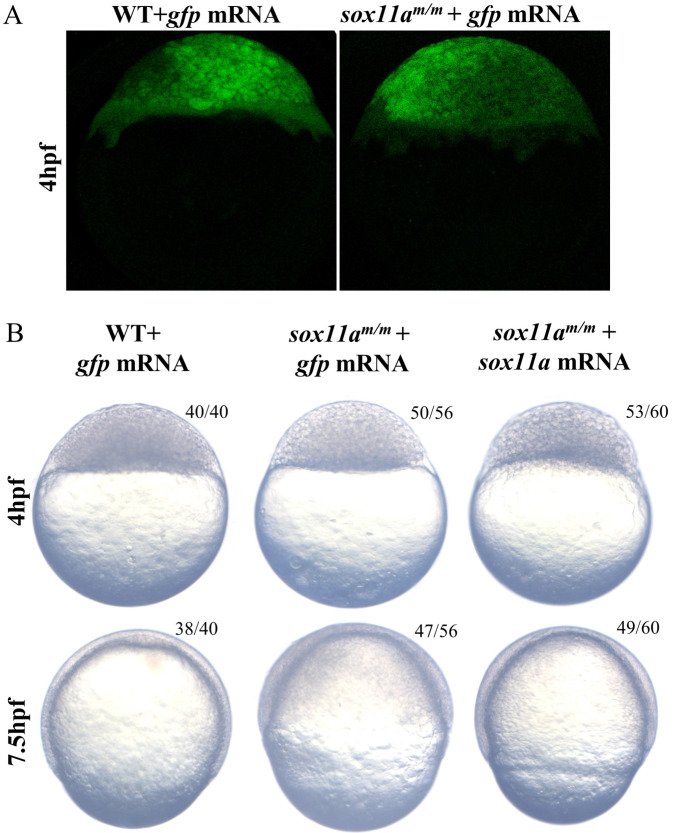
Partial rescue of development delay by *sox11a* mRNA injection. **A:** The *sox11a^m/m^*mutant and WT could produce green fluorescence after injection of *gfp* mRNA, which indicated the microinjection was effective. **B:** The *sox11a^m/m^*mutant was rescued by *sox11a* mRNA while the injection of *gfp* mRNA as controls. The *sox11a^m/m^*mutant injected with zebrafish *sox11a* mRNA could be partially rescued at 4 hpf and 7.5 hpf.

To confirm whether the phenotype was caused by the disruption of *sox11a*, the *sox11a^m/m^* mutants were rescued by the injection of *sox11a* mRNA, while the two control groups were set by injection of exogenous *gfp* mRNA (Fig. 5A). The results showed that *sox11a^m/m^* mutants injected with zebrafish *sox11a* mRNA developed significantly faster than the mutant group injected with *gfp* mRNA, but its process was still a little slower than that of the WT group injected with *gfp* mRNA. At 7.5 h after injection, we observed that the* sox11a^m/m^* mutants injected with *sox11a* mRNA developed into 65% epiboly stage, while the *sox11a^m/m^* mutants and WTs injected with *gfp* mRNA developed into 50% and 75% epiboly, respectively (Fig. 5B).

### Targeted disruption of s*ox11a* in zebrafish affects the expression of genes related to cartilage and bone development

**Figure 6:**
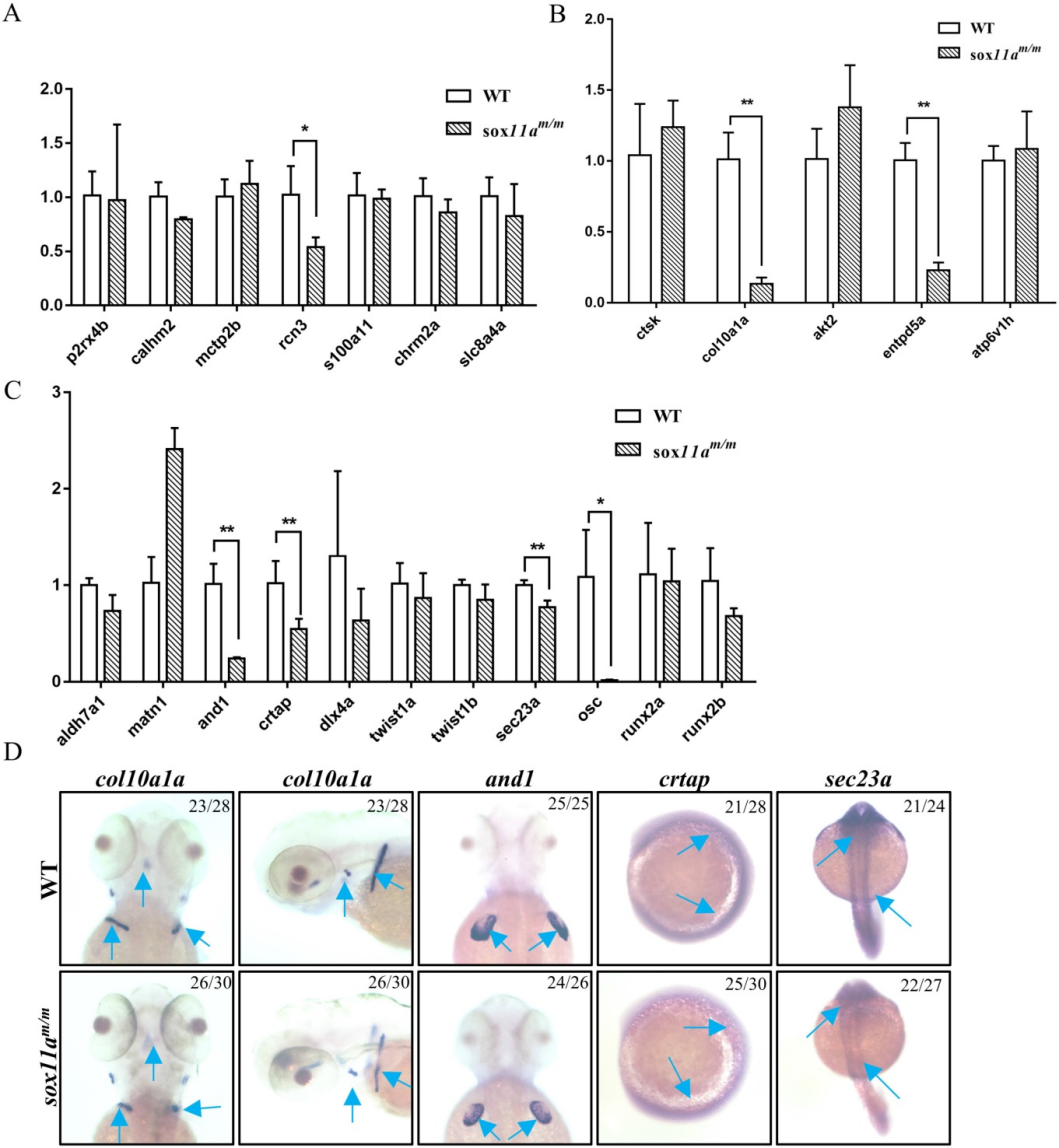
Analysis of the expression of genes related to skeleton development by qPCR and whole-mount *in situ* hybridization. **A:** Expression profile of genes involved in calcium signaling pathway (*P<0.05). **B:** Expression profile of genes involved in cartilage development (**P<0.01). **C:** Expression profile of genes involved in bone development (*P<0.05, **P<0.01). The qPCR data analysis use by 2^-∆∆CT^ and *ꞵ-actin* was chosen as reference gene. **D:** Analysis of the expression of genes involved in skeleton development by whole-mount *in situ* hybridization staining for zebrafish *col10a1a*, *and1*, *crtap* and *sec23a* during embryogenesis.

To further elucidate the mechanism of disruption of *sox11a* affecting skeleton development, the expression level of genes related to calcium signaling pathway, cartilage development, and bone development in adult zebrafish were examined by qPCR. We found no significant changes in the calcium signaling pathway, except for a slight decrease in *rcn3* expression levels in *sox11a^m/m^* mutant zebrafish (Fig. 6A). In the bone development-related pathway, we detected significant downregulation in the expression levels of *col10a1a* and *entpd5a* (Fig. 6B). Moreover, there were also significantly lower expression levels of *and1*,* crtap,* and *sec23a* in the *sox11a^m/m^* mutant, which is involved in cartilage development (Fig. 6C). Furthermore, the expression levels of *col10a1a*, *and1*, *crtap,* and *sec23a* were checked in embryos by whole-mount *in situ* hybridization. The results indicated that the expression levels of these genes were all downregulated, which is consistent with the qPCR experimental results (Fig. 6D). These data suggested that skeletal dysplasia in *sox11a^m/m^* mutant zebrafish was mainly a result of the abnormal expression levels of skeletal-related signaling pathways.

## Discussion

In the present study, *sox11a^m/m^* and* sox11b^m/m^* mutant zebrafish were generated via CRISPR/Cas9 for the first time. The results herein obtained suggested that the *sox11a^m/m^* mutant zebrafish could survive and develop into adults but display growth and developmental delay, besides microcephaly with typical Coffin-Siris syndrome characteristics. However, no differences were found between the *sox11b^m/m^* mutant zebrafish and WT zebrafish.

### The *sox11a^m/m^* mutant zebrafish possessed characteristics of human Coffin-Siris syndrome

In clinical statistics, human Coffin-Siris syndrome is related to the mutation of *SOX11* 7. However, *Sox11* homozygous mutant mice normally die soon after birth 10, which hinders the study of the pathogenic mechanism of Coffin-Siris syndrome using mice as models. Several researchers have attempted to build disease models using the morpholino technique to knock down *Sox11* for further research. Hempel et al. used morpholino knockdown *Sox11* in *Xenopus laevis*, which resulted in a significant reduction in head area and interpupillary distance compared with controls 7. Tsurusaki et al. injected *sox11a* or *sox11b* morpholino into zebrafish embryos, and the injected embryos had smaller heads and significantly higher mortality rates compared with the control embryos 8. Nevertheless, it is known that morpholino itself is somewhat toxic and can therefore affect the embryo development 13. In addition, the morpholino technique is only suitable for knockdown of target genes during embryonic development. Therefore, neither MO *Xenopus laevis* nor MO zebrafish are ideal models for Coffin-Siris syndrome.

We herein generated heritable *sox11a^m/m^* and *sox11b^m/m^* mutants using CRISPR/Cas9. The homozygous mutant was obtained by self-crossing, and this method was able to eliminate the maternal effect and produce maternal and zygotic deficiency mutant lines. The *sox11a^m/m^* mutant zebrafish obtained had growth deficiency, pectoral fin development delay, and microcephaly; these features are characteristic of human Coffin-Siris syndrome. Some* sox11a^m/m^* mutants even had a deformed skeleton, which was clearly observed in the Micro-CT scans; however, this effect was not obvious in juvenile fish under 20 dpf. Unlike the lethal effect of *Sox11* knockout in mice, the *sox11a^m/m^* mutant and *sox11b^m/m^* mutant zebrafish herein obtained were viable. This is the first time that viable *sox11* mutants have been obtained in all animals. Thus, the *sox11a^m/m^* mutant zebrafish may be helpful for finding the target gene of *sox11* and it also is an adequate model for further studying the pathogenic mechanisms of Coffin-Siris syndrome.

### The genes related to cartilage and bone development were downregulated, which affected the development of the *sox11a^m/m^* mutant

Skeletons and muscles are of vital importance in body shape development. The length of bones and the number of muscles play a decisive role in human height and weight. Many growth-related diseases in humans are caused by abnormal skeletal and muscular development. Zebrafish skeletal development patterns conform to classical skeletal development patterns 14. The process of skeletal development is easier to observe in zebrafish than in mammals. Therefore, many human skeletal disease models have been established based on studies on zebrafish 15. For example, the *sec23a* mutation in humans causes growth delay, and its mutation in zebrafish causes a shorter body length, which has been used as a disease model for humans 16-18.

The *sox11a^m/m^* mutant zebrafish presented growth and developmental delay, and this phenomenon could last to adulthood. Muscle development in the *sox11a^m/m^* mutants presented no obvious abnormalities such as in the number of myoblast nuclei and the arrangement of muscle fibers (Fig. 4F, G). Since the skeleton is another important factor affecting growth in development, *sox11a^m/m^* mutant skeletal development was subsequently examined (Fig. 4H, I). Alcian blue staining indicated that cartilage development in 5 dpf *sox11a^m/m^* mutant was slower than in WTs. Moreover, the analysis of the Micro-CT scans suggested that some 20 dpf mutants developed bone malformations. Hence, we believe that the defects in the *sox11a^m/m^* mutants were mainly related to skeleton development. We further found that the expression levels of genes related to cartilage development were significantly downregulated (Fig. 6). A previous study showed that *and1* was mainly related to zebrafish fin regeneration 19. Other studies showed that disruption of *sec23a* in zebrafish leads to a shorter body length 20 and that *crtap* plays an important role in bone formation 21. In addition, bone development-related genes, including *col10a1a* and *entpd5a,* were significantly downregulated. As previously reported, *col10a1a* was considered to be related to osteoblast development 22, whereas *entpd5a,* which is expressed in zebrafish osteoblasts, was considered as being related to skeletal mineralization 23. These data suggest that *sox11a* mainly affects the growth and development of zebrafish by regulating the expression of genes related to skeleton development.

The *sox11b^m/m^* mutant zebrafish didn't show any phenotype including growth or development defects. Nevertheless, this did not mean that we failed to disrupt *sox11b* or the *sox11b* was unfunctional. we had detected the down-regulated of *sox11b* in *sox11b^m/m^* mutant by qPCR and whole-mount *in situ* hybridization, which may be mediated by nonsense-mediated mRNA decay 24,25. The expression of *sox11a* in *sox11b^m/m^* mutant was lower than WT (Fig. S6), and the expression of *sox11b* in *sox11a^m/m^* mutant was also lower than WT (Fig. S7). Based on our data, we inferred the *sox11a* and *sox11b* may function together. Moreover, the process of generating *sox11a^m/m^sox11b^m/m^* double mutant further indicated the double mutant was associated with a more severe phenotype than either of the single* sox11a^m/m^* or *sox11b^m/m^* mutants. There was only one copy of the gene in mice, human and *Xenopus laevis* 7,9,10. In particular, the homozygote *Sox11* in mice lead to embryo lethality 10, which was resemble to the double mutant of *sox11a* and *sox11b* in zebrafish.

In conclusion, the disruption of *sox11a* affects skeleton development, which leads to the* sox11a^m/m^* mutant possessing a series of Coffin-Siris syndrome features. To the best of our knowledge, this is the first study to analyze the function of *sox11* by establishing a *sox11a^m/m^* mutant model in vertebrates. Moreover, the *sox11a^m/m^* mutant zebrafish is expected to be a disease model of Coffin-Siris syndrome, which may be expedient to study the pathogenesis mechanism and identify target drugs for this syndrome.

## Supplementary Material

Supplementary figures and tables.

## Abbreviations

dpf: days post fertilization
hpf: hours post fertilization
WT: wild type
MO: morpholino
