# Supplementary material for: Multiple Developmental Defects in sox11a Mutant Zebrafish with Features of Coffin-Siris Syndrome
Source: Int J Biol Sci. 2020 Oct 3;16(15):3039–49. doi: 10.7150/ijbs.47510 (PMC7545714; doi:10.7150/ijbs.47510)

Figure S1: Primer sequence

Primer name	Primer sequence（5‘-3’）	Primer function
sox11a-g1-F	TGTAATACGACTCACTATA ggtcgctttatgtgtccgg GTTTTAGAGCTAGAAAT	gRNA synthesis
sox11a-g2-F	TGTAATACGACTCACTATA ggctgtcttgcaccagtct GTTTTAGAGCTAGAAAT	
sox11b-g1-F	TGTAATACGACTCACTATA gggtcgtttgatgtggccgg GTTTTAGAGCTAGAAAT	
sox11b-g2-F	TGTAATACGACTCACTATA gggtcgtttgatgtggccgg GTTTTAGAGCTAGAAAT	
gRNA-R	AAAAAAAGCACCGACTCGGTGCCAC	
sox11a-F	ACTTCGCCTCCTCCGCGCAA	real-time PCR
sox11a-R	AGCCCAGGCTGCCCTCGCTA	
sox11b-F	ATGGCTGACTACCCCGACT	
sox11b-R	GCTGCTTTGACACTTTTGC	
p2rx4b-F	CTTTGGAATCCGCTTTGACG	
p2rx4b-R	AGGTTAAGAAGGGCAAGAGC	
calhm2-F	GTGATTGAACGCCAGCTACA	
calhm2-R	TATGCCTCCTGCTGATAGCC	
mctp2b-F	CTCCAGAGTGCCCTCAGATA	
mctp2b-R	GCACCTTCACACAGACTGAT	
rcn3-F	ATAATGAAGCCCGGCATCTC	
rcn3-R	TTTGGTGAGATCCTCTCCGT	
s100a11-F	ACAAGCTTGGTCAAATCCCA	
s100a11-R	AGAACTCCATGAACGTCAGC	
chrm2a-F	TGAACGCCAGCTACAGTATG	
chrm2a-R	TCCTGCTGATAGCCAAGAGT	
slc8a4a-F	AAGAAGAGGTGGCCAAGATG	
slc8a4a-R	CAGCGCAAGGTTAGTCTTCT	
aldh7a1-F	GAAGAGTGTTCCGCTGGTTA	
aldh7a1-R	CAAACGCTCCTCCAATCTCA	
matn1-F	TTGCCAAGAAGCTGCAAATC	
matn1-R	CTCTTCGTCACAGCTTCCAA	
and1-F	CTGATCCGCAACAGGAGAAA	
and1-R	CTCTGCAACTCCGTCTTTGT	
crtap-F	AAATTTGGCCTCGACGATGA	
crtap-R	GGCATGAACTTCTGTTTGGC	
dlx4a-F	ATCCAGGAGCTTACCTACCC	

dlx4a-R	CGTTCAGCCGTATTTCTCCA	
twist1a-F	GTCAACATCCCACTAACGCA	
twist1a-R	CTCCTTCCAGTGAGTTCAGC	
twist1b-F	TTCTCGGTTTGGAGGATGGA	
twist1b-R	AGCTCACGGTTTGACCATTT	
sec23a-F	CCCGAGTATGAGAACTTCCG	
sec23a-R	CGTGCTCAGTGTCGATGTAG	
osc-F	ATCAGCTGACACAGAAGCGA	
osc-R	GGCGGTGATGATTCCAGACG	
runx2a-F	GACCATGGTGGAGATCATAGC	
runx2a-R	GGGTTCGTGAATACTGTGATTG	
runx2b-F	AGAGCTTCACCCTGACGATTAC	
runx2b-R	AGGTACGATGGGTATGTCTGGT	
ctsk-F	GTAACGAGAGGGCACTGAC	
ctsk-R	TTCCTTGTTGCAGTTTGGGT	
entpd5a-F	TGAAGAGTGGAGCTTTGGTG	
entpd5a-R	GATGCTGCTTCCTTTGACCT	
col1a1a-F	TCTGCTGGATCAGCTGGTAA	
col1a1a-R	CAATTTCTCCATTGCGACCAC	
akt2-F	AAGAAGCTCGTTCCACCCTT	
akt2-R	GGTCTGTGCAGTGAACTCAT	
atp6v1h-F	CAGGTTATTGCCGTCGCA	
atp6v1h-R	TGTTTACCACCCAGCTGTTC	
β-*actin*-F	ATGCCCCTCGTGCTGTTTTC	
β-*actin*-R	GCCTCATCTCCCACATAGGA	
Probe-*and1*-F	GATGTACCTGCAGCACCTTG	Probe synthesis
Probe-*and1*-R	TAATACGACTCACTATAGGG CATACCCGACGAAACATTCA	
Probe-*crtap*-F	CTTTTCCGTTTGCGTCC	
Probe-*crtap*-R	TAATACGACTCACTATAGGG ACTGCGTTCTTCAGGTCATT	
Probe-*sec23a*-F	ACCAGCCTGCTGAGCTACTT	
Probe-*sec23a*-R	TAATACGACTCACTATAGGG TCCCTGCTAATGCCATTTTA	
Probe-*col10a1a*-F	ACCAGCCTTACTCCGTGAAA	
Probe-*col10a1a*-R	TAATACGACTCACTATAGGG TCCAGGTTTCCCTGAAGGTC	
sox11a-HMRA-F1	TGATGAAAGCGAATTCATGG	Genotyping
sox11a-HMRA-R1	CTGGAGACTGTTCCATGATC	
sox11b-HMRA-F1	CGAGGAGAGCGAAATGATGGCTTG	
sox11b-HMRA-R1	CGCATTCATGGGTCGTTTGATGTG	

Figure S2: The conservation of *sox11* in various species.

```
zb sox11a          QTDNS TD.SMS EAT SD S F VS.......IN       T                                                   82
zb sox11b          QTDHS TESSVS ETT TE S M ACSPVP...PK       T                                                   87
medaka sox11a      HMDNS TDGSMS EAT TE S F ACSPVA...IN       T                                                   87
rat sox11          QAESS AESNLP DAL TE G F ACSPVALDESD       S                                                   90
mice sox11         QAESS AESNLP DAL TE G F ACSPVALDESD       S                                                   90
human sox11        QAESL AESNLP EAL TE G F ACSPVALDESD       S                                                   90

zb sox11a                        K            K    L SSS PSAP.  E CSKT............S S....S   P L ANKTGSKSS   155
zb sox11b                        Q            K    L SSS PAVQ.  E ISKS............V AAA..G   A L PSKPGNITA   162
medaka sox11a                    K            K    L AS. SSAP.  E CAKL............A TP...S   S M K..SGSKS.   157
rat sox11                        K            R    T PAA PSAGQ  D SAAG............A AAKGPG   A L APAG...KA   165
mice sox11                       K            R    T PAA PSAGQ  D SAAG............A AAKGPG   A L APAG...KA   165
human sox11                      K            R    M PSA PSASQ  E SAAGGGGGSAGGGAGGA TSKGSS   G L APAAAGAKA   180

zb sox11a       ...SHGYGDEYAFK..................ST VSKTVHIKSEFTDEDD  DSEEDSRVRVKEEEEDP.................IRA   207
zb sox11b       RASTQDCRFNYVFT..................NL VTKS..IKRELTDDED  DDDDDDDDEEDDYEDE...................EH   213
medaka sox11a   ...AHSYGDDCVFK..................VA T.....VKSELTDDED  DYEEDYRMGIKRAEEER.................LRP   204
rat sox11       GAGKAAQPGDCG....................AG AAKCVFLDDDDEEDDE  ELQLRPKPDADDDDDEPAHSHLLPPPAQQQPPQLLRR   235
mice sox11      GAGKAAQPGDCA....................AG AAKCVFLDDDDEDDDE  ELQLRPKPDADDDDDEPAHSHLLPPPTQQQPPQLLRR   235
human sox11     GAGKAAQSGDYGGAGDDYVLGSLRVSGSGGGGAG TVKCVFLDEDDDDDDD  ELQLQIKQEPDEEDEEPPHQQLLQPPGQQ.PSQLLRR   269

zb sox11a       YNVAK         ST  E.   M E V .........NN  Y NF N.ITKQSTMY...   S........VSPASSRSV T S SS  A   275
zb sox11b       IRLHN         AA  EH   M E S HTSATH...GS  F NF N.ITKQSAAY...   S......VSPASSFRSVS S S SS  S   290
medaka sox11a   YNVAK         SA  E.   M E V S........HN  F NI SKQSAAASVS...   SSRSVSTSSSSSSSSSS S S SG  A   282
rat sox11       YSVAK         AA  PE   L D V AGG........  Y SF N.ITKQQPPP.AP   LSPASSRCVSTSSSS..G S G GA  A   313
mice sox11      YSVAK         AA  PE   L D V AGG........  Y SF N.ITKQQPPP.AP   LSPASSRCVSTSSSS..G S G GA  A   313
human sox11     YNVAK         SA  PE   L D V AGATSGAGGGS  Y SF N.ITKQHPPPLAQ   LSPASSRSVSTSSSSSSG S G SG  A   358

zb sox11a          LF F   FASSAQSS...E  SQ.NP   S      E E                                                  353
zb sox11b          LV F   LAAGSHTA...D  .N.TS   C      D D                                                  367
medaka sox11a      LF F   FAPSAPGS...E  ...NS   S      D D                                                  358
rat sox11          MF L   FSQGAHSACEQP  AG.AA   S      D D                                                  394
mice sox11         MF L   FSQGAHSACEQP  AG.AA   S      D D                                                  394
human sox11        MF L   FSQSAHSASEQQ  GGAAA   S      D D                                                  440
```

Figure S3: The second line of *sox11a*$^{m/m}$ mutant and *sox11b*$^{m/m}$ mutant

A: The target site of *sox11a*$^{m/m}$ mutant line.

CTCGATAAACCCAGACTGGTGCAAGACAGCCACCGGACACATA

CTCGATAAACCCAGAC--------------AGCCACCGGACACATA -11bp

B: The amino sequence of Sox11a

M V Q Q T D N S E T D S M S R E A T D S D E S E F M V S I N P D W C K T A T
G H I K R P M N A F M V W S K I E R R K I M E Q S P D M H N A E I S K R L G
K R W K M L K D S E K I P F I R E A E R L R L K H M A D Y P D Y K Y R P K K
K P K L D S S S K P S A P S P E K C S K T S K S S K K C P K L K A N K T G S K
S S S H G Y G D E Y A F K S T K V S K T V H I K S E F T D E D D D D D S E E D
S R V R V K E E E E D P I R A Y N V A K V P A S P T L S S S T E S E G A S M Y E
E V R N N R L Y Y N F K N I T K Q S T M Y P A S V S P A S S R S V S T S S S S S
E D A D D L L F D F S L N F A S S A Q S S E L G S Q N P G N L S L S L V D K E
L E S F S E G S L G S H F E F P D Y C T P E L S E M I A G D W L E A N F S D L V
F T Y *

C: A frameshift mutation of *sox11a*$^{m/m}$ mutant

M V Q Q T D N S E T D S M S R E A T D S D E S E F M V S I N P D S H R T H K
A T D E R V H G V V *

D: The target site of *sox11b*$^{m/m}$ mutant line.

GGTGCCACCGAAACCGGACTGGTGCAAGACAGCCACCGGCCACAT

GGTGCCACCGAAACCG--ACTGGTGCAAGACAGCCACCGGCCACAT -1bp

E: The amino sequence of Sox11b

M V Q Q T D H S E T E S S V S R E T T D T E E S E M M A C S P V P P K P D W
C K T A T G H I K R P M N A F Met V W S K I E R R K I M E Q S P D M H N A E I
S K R L G K R W K M L K D S E K I P F I R E A E R L R L Q H M A D Y P D Y K
Y R P K K K P K L D S S S K P A V Q S P E K I S K S V K A A A G K K C A K L K
P S K P G N I T A R A S T Q D C R F N Y V F T N L K V T K S I K R E L T D D E
D D D D D D D D D D D E E D D Y E D E E H I R L H N V P A S P T L S S A A E S
E H G A S M Y E E S R H T S A T H G S R L F Y N F K N I T K Q S A A Y P A S V
S P A S S F R S V S S S S S S S S E D S D D L L V D F S L N L A A G S H T A D L
G N T S G N L C L S L V D K D L D S F S E G S L G S H F E F P D Y C T P E L S
E M I A G D W L E A N F S D L V F T Y *

F: A frameshift mutation of *sox11b*$^{m/m}$ mutant

M V Q Q T D H S E T E S S V S R E T T D T E E S E **M M** A C S P V P P K P T G A
R Q P P A T S N D P *

Figure S4: The production process of *sox11a*$^{m/m}$*sox11b*$^{m/m}$ double mutant.

sox11a$^{+/+}$*sox11b*$^{+/+}$	*sox11a*$^{+/+}$*sox11b*$^{m/+}$	*sox11a*$^{m/+}$*sox11b*$^{+/+}$	*sox11a*$^{m/+}$*sox11b*$^{m/+}$
sox11a$^{+/+}$*sox11b*$^{m/+}$	*sox11a*$^{+/+}$*sox11b*$^{m/m}$	*sox11a*$^{m/+}$*sox11b*$^{m/+}$	*sox11a*$^{m/+}$*sox11b*$^{m/m}$
sox11a$^{m/+}$*sox11b*$^{+/+}$	*sox11a*$^{m/+}$*sox11b*$^{m/+}$	*sox11a*$^{m/m}$*sox11b*$^{+/+}$	*sox11a*$^{m/m}$*sox11b*$^{m/+}$
sox11a$^{m/+}$*sox11b*$^{m/+}$	*sox11a*$^{m/+}$*sox11b*$^{m/m}$	*sox11a*$^{m/m}$*sox11b*$^{m/+}$	*sox11a*$^{m/m}$*sox11b*$^{m/m}$

Figure S5: The ratio of *sox11a*$^{m/m}$ mutant with curved spine (***P*<0.0001).**

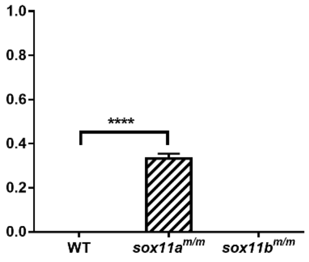

Figure S6: The expression level of *sox11a* in WT and *sox11b*$^{m/m}$ mutant (P*<0.05).**

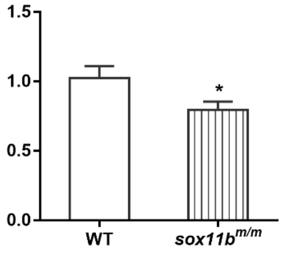

Figure S7: The expression level of *sox11b* in WT and *sox11a*$^{m/m}$ mutant (*P*<0.01).**

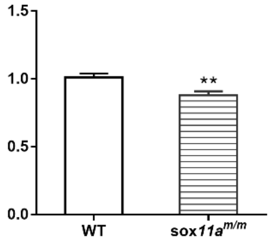

Supplement: Supplementary file 1 — Supplementary figures and tables. [file ijbsv16p3039s1.pdf]
